# Evaluation of Patient-Centered Outcomes Associated With the Acceleration of en-Masse Retraction of Upper Anterior Teeth Assisted by Flapless Corticotomy Compared to Traditional Corticotomy: A Two-Arm Randomized Controlled Trial

**DOI:** 10.7759/cureus.42273

**Published:** 2023-07-21

**Authors:** Hanin N Khlef, Mudar Mohammad Mousa, Ali Mohsen Ammar, Mohammad Y Hajeer, Mohammed Adel Awawdeh

**Affiliations:** 1 Department of Orthodontics, Faculty of Dentistry, University of Damascus, Damascus, SYR; 2 Department of Orthodontics, Faculty of Dentistry, Arab Private University for Science and Technology, Hama, SYR; 3 Department of Preventive Dental Science, College of Dentistry, King Saud Bin Abdulaziz University for Health Sciences, Riyadh, SAU

**Keywords:** functional impairment, orthodontic mini-screws, flapless corticotomy, traditional corticotomy, accelerated tooth movement, extraction, class ii division 1 malocclusion, en-masse retraction, discomfort, pain

## Abstract

Objective: This study aimed to assess the levels of pain, discomfort, and functional impairment associated with the en-masse retraction of the upper anterior teeth when treating Class II division 1 malocclusion patients using traditional corticotomy or flapless corticotomy. In addition, an assessment of patients' satisfaction with the selected surgical intervention was undertaken at one-month post-operatively.

Materials and methods: The study sample comprised 40 patients with Class II division 1 malocclusion, randomly assigned to either the traditional corticotomy group (n=20) or the flapless corticotomy group (n=20). Patients underwent extraction of the maxillary first premolars, and orthodontic mini-screws were placed between the maxillary second premolars and the first molars for skeletal anchorage. An en-masse retraction was accomplished in both groups. Patients were asked to fill in a questionnaire at 24 hours (T1), four days (T2), seven days (T3), 14 days (T4), and 28 days (T5) after the surgical intervention using standardized questionnaires. Most questions were answered on a visual analog scale where zero scores meant the absence of pain, discomfort, or functional impairment, and 100 scores meant the worst feelings of these traits.

Results: All patients in both groups entered data analysis with no dropouts. All measured levels were significantly greater in the traditional corticotomy group during the first two weeks following the corticotomy intervention in terms of pain perception (P˂0.001), discomfort (P=0.004), and difficulty in chewing (P=0.015). Additionally, during the first week following corticotomy, levels of perception of discomfort (P˂0.001), difficulty in swallowing (P=0.001), and limitation of jaw movement (P˂0.001) were significantly greater in the traditional corticotomy group. Patient satisfaction, the recommendation to a friend, and acceptance of flapless corticotomy were significantly greater than traditional corticotomy (P=0.002, P=0.001, respectively). 78% of patients in the traditional corticotomy group considered it more discomfort than a tooth extraction, while 50% of patients in the flapless corticotomy group considered tooth extraction more discomfort, with a significant difference between the two groups (P=0.001).

Conclusions: The levels of negative patients’ reported outcomes were significantly smaller with flapless corticotomy than with traditional corticotomy. Traditional corticotomy was associated with mild to moderate levels of pain, swallowing difficulty, moderate levels of discomfort, chewing difficulty, and jaw movement limitation after 24 hours of the surgical procedure. In contrast, flapless corticotomy was less problematic and associated with mild pain, swelling, chewing difficulty, jaw movement limitation, and swallowing difficulty at the same assessment time. Patient satisfaction, acceptance, and recommendation to a friend were greater for flapless corticotomy than traditional intervention.

## Introduction

Prolonging the duration of orthodontic treatment can lead to adverse effects such as dental cavities, periodontal diseases [1], and root resorption [2]. Therefore, various adjunctive therapeutic procedures have emerged to reduce treatment time [3,4]. These include low-level laser stimulation [5], surgical methods such as corticotomy [6], and periodontally accelerated osteogenic orthodontics [7]. Retraction of the upper anterior teeth is considered an important stage in orthodontic treatments, accompanied by the extraction of dental units with the aim of camouflaging Class II malocclusion [8]. Many studies have indicated that the en-masse retraction of the upper anterior teeth is preferable to two-stage retraction due to its multiple biomechanical advantages [9], particularly in shortening orthodontic treatment time [10].

Considering that most of the negative aspects associated with using surgical methods to accelerate orthodontic tooth movement result from using traditional surgical instruments such as burs [11], the search for less invasive surgical instruments has been pursued [12]. However, the traditional corticotomy group (TCG) is considered invasive due to the need for raising a gingival flap and requiring surgical sutures [3]. Therefore, there has been increasing interest in developing more conservative surgical techniques, including flapless corticotomy group (FCG) using a piezosurgical device [13,14], corticision [15], laser-assisted flapless corticotomy [16,17], and low-intensity direct electrical current [18]. These procedures do not require surgical intervention with flap elevation.

Orthodontic treatment carries some unwanted side effects like any other medical treatment, with pain and discomfort being the most common [19,20]. The fear of pain is one of the main reasons patients avoid orthodontic treatment, and pain is classified as the primary reason for discontinuing orthodontic treatment [21]. Pain and discomfort in patients during orthodontic treatment can be assessed using reliable pain assessment scales, such as the visual analog scale (VAS) or the numerical rating scale (NRS) [22]. According to a recent systematic review, it is impossible to obtain clear results for pain and discomfort associated with piezocision-assisted corticotomy in orthodontic en-masse retraction cases due to the presence of only one study [23]. Additionally, the strength of evidence related to patient-centered outcomes ranged from weak to very weak. Consequently, it can be concluded that further high-quality controlled studies are required [23]. Considering the scarcity of studies in the medical literature on this topic, this trial was conducted. Therefore, the objective of this study was to assess the levels of associated pain and discomfort when retracting the anterior upper six teeth using traditional or flapless corticotomy techniques and to compare the two methods.

## Materials and methods

Study design and settings

This study employed a controlled clinical trial design using parallel groups. The orthodontic treatment was performed at the Department of Orthodontics and Dentofacial Orthopedics at the University of Damascus by the first author (Hanin N. Khlef) under the supervision of Mohammad Y. Hajeer. Both surgical interventions (traditional corticotomy and flapless corticotomy) were carried out at the Department of Oral and Maxillofacial Surgery at the University of Damascus by the first author (Hanin N. Khlef) under the supervision of a Professor of Oral and Maxillofacial Surgery (Omar Heshmeh). This study was registered in the ClinicalTrials.gov database (NCT05928143). The funding was provided by the University of Damascus Postgraduate Research Budget (Ref no: 35632563781JJA). The ethical approval was obtained from the Local Research Ethics Committee of the University of Damascus (Reference number: UDDS-998-10012019/SRC-234).

Sample size estimation

Minitab® Version 17 (Minitab Inc., State College, Pennsylvania, USA) software was used to estimate the sample size. A significance level of 5% and a power of 85% were selected, assuming that the minimum clinically significant difference requiring detection between the two groups in pain level was 25 mm on the VAS with a standard deviation of 23.73 mm [24]. Therefore, the required number of patients for each group was estimated to be 18, resulting in a total sample size of 36. Assuming a withdrawal rate not exceeding 10% during the follow-up period, the total required sample size for the study was 40 patients, i.e., 20 patients in each group.

Patient recruitment

**Figure 1:**
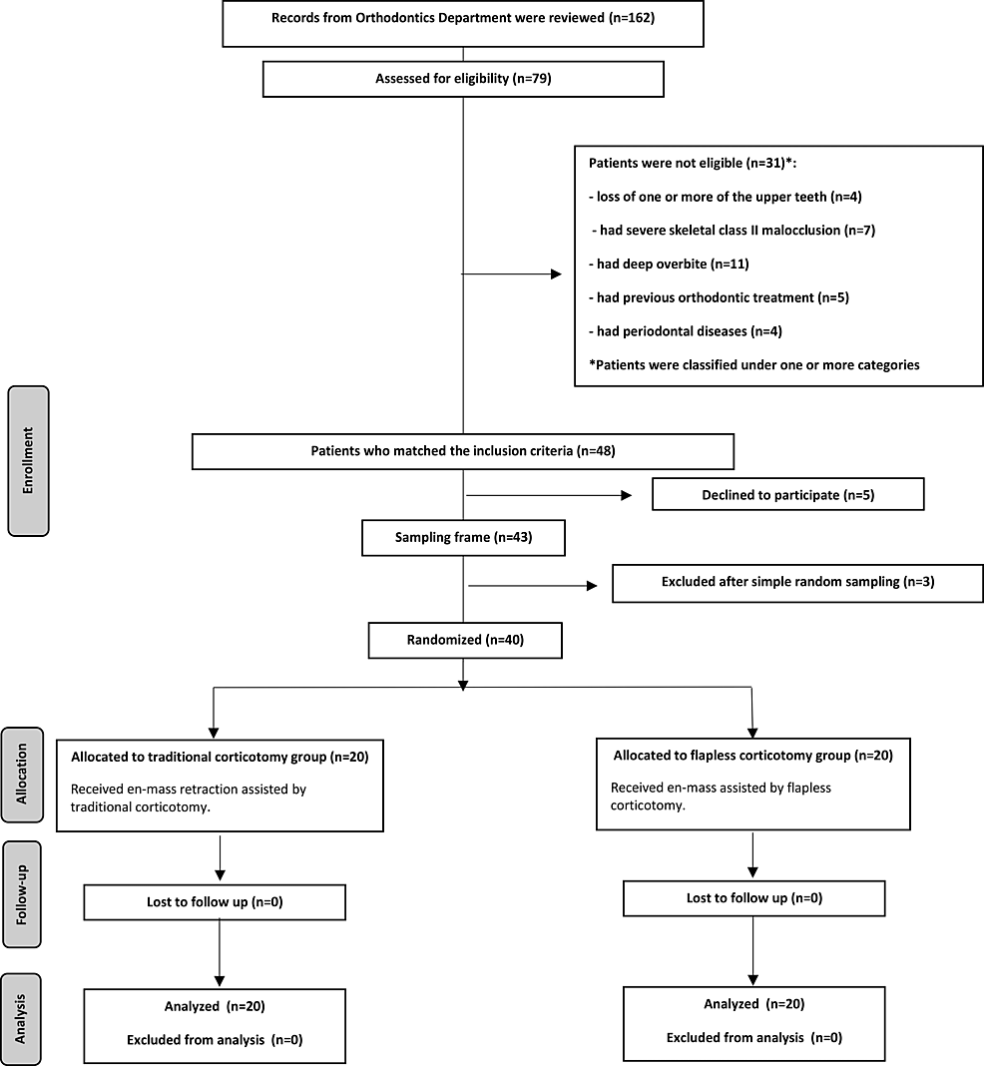
Flow diagram of patient recruitment, assignment, follow-up, and entry into data analysis.

A review of the Department of Orthodontics records at the Faculty of Dentistry, Damascus University, was conducted. The follow-up of patients referred to the department was also done from February 2018 to May of the same year to complete the sample construction. After a clinical examination of 79, the number of patients strictly met the inclusion criteria was 48. The eligible patients were informed about the study details, and a detailed explanation of the two treatment methods used was provided to them. An information sheet was given to the patients, and all their questions were answered. Informed consent was obtained from 45 patients, while four patients declined to undergo surgical intervention as part of their orthodontic treatment; 40 patients were randomly selected (Figure 1).

The inclusion criteria were: adult patients aged between 18 and 30 years, Class II division I malocclusion, normal or increased vertical dimension (confirmed radiographically through cephalometric angles: the angle between the maxillary plane and the mandibular plane (MM), the angle between the anterior cranial base and the mandibular plane (MP-SN), and the angle between the anterior cranial base and the facial axis (Y-axis)), mild to moderate skeletal Class II relationship, clinically assessed, and later confirmed radiographically through cephalometric angle skeletal relationship in the midsagittal plane (ANB), lower incisor coverage equal to or less than one-third of the vertical height of upper incisors and protrusion ranging from 5 to 10 mm, all permanent teeth present in the maxilla regardless of third molars, no crowding, and if present, it should be less than 3 mm, the presence of healthy supportive tissues and good oral health, clinically evaluated through periodontal probing.

The exclusion criteria were patients with contraindications that prevent oral surgery under local anesthesia (social, psychological, medical), patients with general health conditions that affect dental movement (patients taking non-steroidal anti-inflammatory drugs, corticosteroids, and bisphosphonates, hyperthyroidism and hypothyroidism, uncontrolled diabetes, osteomalacia, and osteoporosis), severe facial, dental syndromes or congenital facial asymmetry, patients who have undergone previous orthodontic treatment, patients with congenital tooth loss or extraction of one of the maxillary permanent teeth (excluding third molars), dental trauma and anomalies or the presence of supernumerary or impacted teeth, patients with poor oral health or active disease around the teeth.

Randomization and patient assignment

A simple computerized randomization method was used, where a faculty member of the Orthodontic Department, who was not involved in this research, created a list of random numbers of patients using Minitab® Version 17 (Minitab Inc., State College, Pennsylvania, USA), with an allocation ratio of 1:1. The sequence of allocation was concealed by placing patients' names and the assigned groups in sealed opaque envelopes, which were not opened until the commencement of the retraction stage of orthodontic treatment. Thus, the sample was divided into two main groups: TCG and FCG. The differences between the two surgical techniques are explained in detail elsewhere [3].

Outcome measures: the questionnaires

The questionnaire was explained in language that the patients easily understood. The patients themselves answered the questions while seated in the treatment chair. The researcher answered any questions without influencing the patient’s responses. Patients were allowed to take Paracetamol 500 mg in case of severe pain after completing the questionnaire to ensure it did not affect the accuracy of the assessment. The study included two questionnaires distributed at multiple time points from the day following the surgical intervention up to 28 days after.

**Figure 2:**
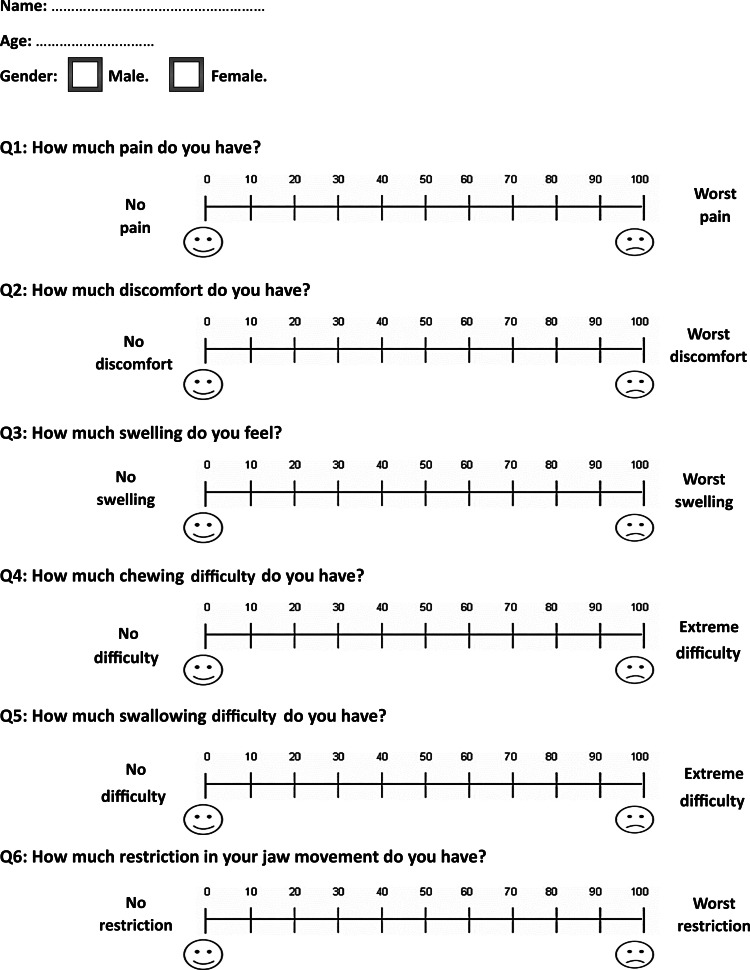
The questionnaire provided to the included patients at all assessment times except for the last assessment time, i.e., at 28 days following the surgical intervention.

The first questionnaire was given to patients at the following assessment times: 24 hours (T1), four days (T2), seven days (T3), and 14 days (T4) after the surgical intervention in both groups. It included six questions regarding the degree of sensation related to (1) pain, (2) discomfort, (3) swelling of the lips and/or cheeks, (4) difficulty in chewing, (5) difficulty in swallowing, and (6) limitation in jaw movement (Figure 2).

**Figure 3:**
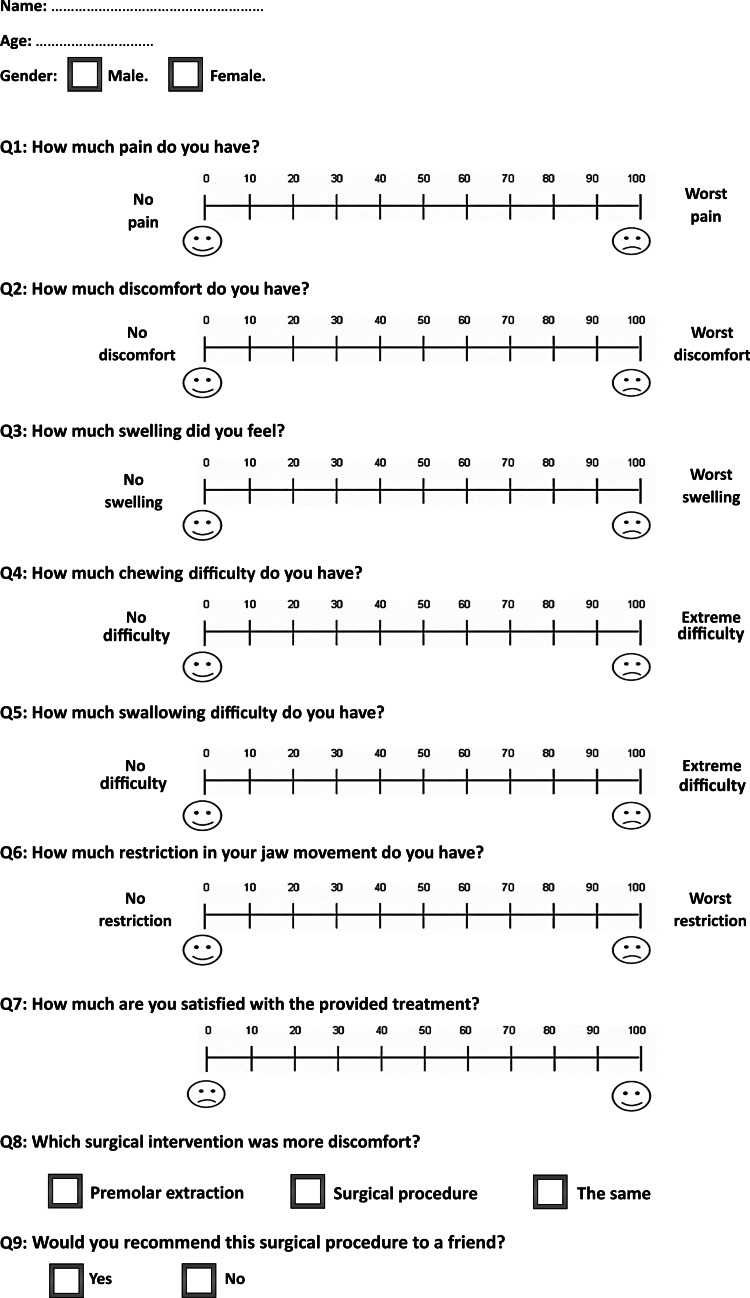
The questionnaire provided to the included patients at the last assessment time, i.e., at 28 days following the acceleratory surgical intervention.

In both groups, the second questionnaire was given to patients at the fifth assessment time (T5), i.e., 28 days after the surgical intervention. It included the questions from the first questionnaire, with an additional three questions regarding: (1) satisfaction with the performed surgical procedure, (2) patient selection of the more discomfort experience during treatment (premolar extraction, the acceleratory surgical intervention, or both), and (3) whether they would recommend the surgical procedure to a friend undergoing orthodontic treatment (Figure 3).

All the questions (except the last two questions of the second questionnaire) were answered using a VAS, a horizontal line measuring 100 mm with two fixed points at the beginning and the end. For example, for the question about the degree of pain, point 0 represented no pain, and point 100 represented the worst possible pain sensation. The wording of the scale was modified to fit the variable under study. The VAS score was then determined by measuring the line length using a ruler, starting from the left side to the mark placed by the patient, representing their current condition. The second-to-last question of the second questionnaire was answered using a three-point scale (premolar extraction/surgical procedure/the same), whereas the last question of the second questionnaire was answered using a binary scale (Yes/No). The severity of each variable was divided into the following categories based on the VAS scores: mild (less than 20), mild-to-moderate (between 20 and 40), moderate (between 40 and 60), moderate-to-severe (between 60 and 80), and severe (between 80 and 100) [23]. An additional clinical outcome was measured in this trial in terms of the surgical time required to conduct the acceleratory intervention in both groups. This outcome was measured in minutes.

Statistical analysis

 Minitab® Version 17 software (Minitab Inc., State College, Pennsylvania, USA) and SPSS ® Version 20 (SPSS for Windows, version 20, IBM Corporation, Armonk, NY) were used to analyze the collected data. The normality of the distributions was assessed using the Anderson-Darling Normality Test. The Mann-Whitney U test was employed to detect significant differences between the two groups, and the one-way repeated measures ANOVA was used to identify significant differences within the same group across different assessment times. As for the last two questions in the second questionnaire, the chi-squared and Fisher's exact tests were employed. The significance level was set at 0.05.

## Results

The sample consisted of 40 patients (36 females and four males), distributed equally into two main groups: the FCG, which included 19 females and one male with a mean age of 22.44±3.55 years, and the TCG, which included 17 females and three males with a mean age of 21.89±3.60 years. There was no statistically significant difference in the mean age between the two groups (P=0.640).

After 24 hours, pain levels were in the “mild to moderate” category, while discomfort and difficulty in chewing were in the “moderate” category in the TCG, with mean values of 27.50, 38.50, and 36.50 mm, respectively. In the FCG group, the pain and difficulty in chewing sensations were at a “mild” level, while discomfort levels were “mild to moderate”; mean VAS values were 14.50, 22, and 18 mm, respectively. The mean values gradually decreased in both groups and approached zero after 28 days. The two groups had statistically significant differences at all assessment times except for the 28th-day evaluation (T5), as shown in Tables 1, 2.

**Table 1:** Descriptive statistics of patient-centered variables of questions 1 and 2 at the five assessment times in the three groups using visual analog scales and the results of the significance testing SD: standard deviation; Min: minimum; Max: maximum; T1: after 24 hours of the beginning of orthodontic treatment; T2: after 4 days; T3: after 7 days; T4: after 14 days; T5: after 28 days; FCG: flapless corticotomy group; TCG traditional corticotomy group; NS: There was no statistically significant difference at P > 0.05. † Employing Mann-Whitney U test. * Statistically significant difference at P < 0.05; ** Statistically significant difference at P < 0.01; *** Statistically significant difference at P < 0.001

	Group	Mean VAS score	SD	Min	Max	P-value†	Significance
Q1: Pain							
T1	FCG	14.10	10.63	0.00	31.00	0.010	*
	TCG	34.85	27.70	1.00	100.00		
T2	FCG	5.40	4.14	0.00	12.00	˂0.001	***
	TCG	31.55	23.87	3.00	95.00		
T3	FCG	3.55	3.35	0.00	10.00	˂0.001	***
	TCG	20.25	21.85	2.00	88.00		
T4	FCG	0.90	1.21	0.00	3.00	˂0.001	***
	TCG	6.25	7.45	0.00	29.00		
T5	FCG	0.30	0.80	0.00	3.00	0.086	NS
	TCG	2.40	6.40	0.00	29.00		
Q2: Discomfort							
T1	FCG	22.90	14.31	1.00	52.00	0.021	*
	TCG	47.60	32.13	1.00	100.00		
T2	FCG	11.60	8.58	1.00	32.00	˂0.001	***
	TCG	40.45	26.19	1.00	100.00		
T3	FCG	6.95	7.25	0.00	22.00	0.004	**
	TCG	25.10	26.19	0.00	100.00		
T4	FCG	1.30	2.58	0.00	11.00	0.004	**
	TCG	6.80	8.94	0.00	38.00		
T5	FCG	0.50	0.76	0.00	2.00	0.344	NS
	TCG	1.90	4.85	0.00	22.00		

**Table 2:** Descriptive statistics of patient-centered variables of questions 3 and 4 at five assessment times in the three groups using visual analog scales and the results of the significance testing SD: standard deviation; Min: minimum; Max: maximum; T1: after 24 hours of the beginning of orthodontic treatment; T2: after four days; T3: after seven days; T4: after 14 days; T5: after 28 days; FCG: flapless corticotomy group; TCG traditional corticotomy group; NS: There was no statistically significant difference at P > 0.05. † Employing Mann-Whitney U test; * Statistically significant difference at P < 0.05; ** Statistically significant difference at P < 0.01; *** Statistically significant difference at P < 0.001.

	Group	Mean VAS score	SD	Min	Max	P-value†	Significance
Q3: Swelling							
T1	FCG	17.35	10.35	0.00	34.00	˂0.001	***
	TCG	70.10	20.42	32.00	100.00		
T2	FCG	6.50	4.21	1.00	15.00	˂0.001	***
	TCG	51.15	22.42	21.00	90.00		
T3	FCG	2.75	3.52	0.00	11.00	˂0.001	***
	TCG	28.60	28.09	0.00	82.00		
T4	FCG	1.30	1.49	0.00	4.00	0.185	NS
	TCG	5.65	9.03	0.00	33.00		
T5	FCG	1.25	1.45	0.00	4.00	0.655	NS
	TCG	2.05	3.94	0.00	12.00		
Q4: Difficulty in chewing							
T1	FCG	18.00	14.15	18.00	14.15	0.003	**
	TCG	45.25	28.69	45.25	28.69		
T2	FCG	8.50	6.35	8.50	6.35	˂0.001	***
	TCG	39.55	25.89	39.55	25.89		
T3	FCG	3.55	3.56	3.55	3.56	0.003	**
	TCG	27.50	30.39	27.50	30.39		
T4	FCG	1.10	1.55	1.10	1.55	0.015	*
	TCG	8.35	11.43	8.35	11.43		
T5	FCG	0.90	1.29	0.90	1.29	0.626	NS
	TCG	0.90	1.94	0.90	1.94		

The swelling sensation after 24 hours in the TCG was moderate to severe, with a mean value of 71.50 mm, while mild in the FCG group, with a mean value of 18.50 mm. There was a statistically significant difference between the two groups during the first week (P<0.001). The values gradually decreased in both groups over time, and the differences became non-significant after 14 days and 28 days P=0.185, P=0.655, respectively. The difficulty in swallowing at the 24th-hour-assessment and fourth-day assessment in the FCG group was closer to zero compared to a mild difficulty level in the TCG, 16 and 10 mm, respectively (Table 3). The limitation in jaw movement in the FCG group was mild after 24 hours and four days of the surgical operation, 7.50 and 5.50 mm, respectively, compared to moderate levels after 24 hours and mild to moderate levels after four days 50, and 33.50 mm, respectively, in the TCG. There was a statistically significant difference between the two groups during the first four days. The values gradually decreased, and the differences became non-significant in subsequent evaluation times.

**Table 3:** Descriptive statistics of patient-centered variables of questions 5, 6, and 7 at the assessment times in the three groups using visual analog scales and the results of the significance testing SD: standard deviation; Min: minimum; Max: maximum; T1: after 24 hours of the beginning of orthodontic treatment; T2: after four days; T3: after seven days; T4: after 14 days; T5: after 28 days; FCG: flapless corticotomy group; TCG traditional corticotomy group; NS: There was no statistically significant difference at P > 0.05. † Employing Mann-Whitney U test; ** Statistically significant difference at P < 0.01; *** Statistically significant difference at P < 0.001.

	Group	Mean VAS score	SD	Min	Max	P-value	Significance
Q5: Difficulty in swallowing							
T1	FCG	2.70	3.08	0.00	12.00	˂0.001	***
	TCG	27.30	29.39	0.00	99.00		
T2	FCG	2.10	2.27	0.00	8.00	0.001	**
	TCG	18.70	23.74	1.00	95.00		
T3	FCG	2.25	2.05	0.00	6.00	0.387	NS
	TCG	8.00	19.48	0.00	89.00		
T4	FCG	0.75	1.37	0.00	4.00	0.291	NS
	TCG	3.85	11.02	0.00	49.00		
T5	FCG	0.50	1.05	0.00	4.00	0.310	NS
	TCG	1.10	1.52	0.00	4.00		
Q6: Limitation in jaw movement							
T1	FCG	9.25	8.40	0.00	22.00	˂0.001	***
	TCG	52.80	25.64	9.00	94.00		
T2	FCG	6.40	5.08	0.00	16.00	˂0.001	***
	TCG	39.70	23.97	6.00	91.00		
T3	FCG	4.15	4.70	0.00	14.00	0.052	NS
	TCG	21.50	25.22	0.00	81.00		
T4	FCG	0.95	1.40	0.00	4.00	0.168	NS
	TCG	7.25	12.38	0.00	44.00		
T5	FCG	0.25	0.64	0.00	2.00	0.156	NS
	TCG	4.40	5.16	0.00	17.00		
Q7: Satisfaction							
T5	FCG	93.75	6.97	79.00	100.00	0.002	**
	TCG	78.55	21.79	10.00	100.00		

Patient satisfaction levels in the FCG group were significantly greater (median value: 97.50 mm) than those of the TCG (median value: 78 mm), with a statistically significant difference between the two groups (P=0.002). About 50% of patients in the FCG group considered the extraction of the first premolars more uncomfortable than the surgical acceleratory procedure, while 78% of patients in the TCG considered the traditional corticotomy procedure more uncomfortable than the extraction of the first premolars, with a statistically significant difference between the two groups (P˂0.001). All patients in the FCG advised a friend to undergo this technique in the context of orthodontic treatment, whereas only 72% of patients in the TCG recommended this intervention, with a statistically significant difference between the two groups (P=0.047) (Table 4).

**Table 4:** Descriptive statistics of patient-centered variables of questions 8 and 9 after 28 days post-surgery in the three groups and the results of the significance testing SD: standard deviation; N: number of patients; FCG: flapless corticotomy group; TCG traditional corticotomy group †Employing Fisher’s Exact Test; * Statistically significant difference at P < 0.05; *** Statistically significant difference at P < 0.001.

		FCG			TCG			P-value†	Significance
Q8: The more discomfort of surgical intervention	Choice	Premolar extraction	Surgical Procedure	The same	Premolar extraction	Surgical Procedure	The same		
	N (%)	10 (50(%	3 (15%)	7 (35%)	2 (10(%	16 (80%)	2 (10%)	<0.001	***
Q9: Recommendation of the procedure to a friend	Choice	Yes	No		Yes	No			
	N (%)	20 (100%)	0 (0%)		15 (75%)	5 (25%)		0.047	*

## Discussion

Pain and discomfort occurred in both corticotomy groups, which could be attributed to soft tissue trauma of the gingiva and alveolar bone during corticotomy. This was accompanied by swelling, difficulty in chewing and swallowing, and limitation of jaw movement in patients. The pain, discomfort, and difficulty chewing levels were significantly higher in the TCG at all evaluation times except after 28 days. This can be explained by the fact that traditional corticotomy involves lifting full-thickness flaps bilaterally from the vestibular and palatal sides and performing vertical and horizontal cortical cuts with surgical suturing, resulting in greater trauma to both bony structures and soft tissues with more severe edema inside and outside the oral cavity [3,6,25]. In contrast, flapless corticotomy was limited to vertical incisions without flap elevation or detachment. Finally, the surgical time for traditional corticotomy ranged from 60 to 75 minutes, while for flapless corticotomy, it ranged from 20 to 30 minutes, with an increase in the surgical time leading to greater subsequent trauma.

The current study differed from Al-Naoum et al.'s on the pain perception variable [6]. In their study, pain perception ranged from mild to moderate in 66% of the sample during the day and was severe in 23.33% of the sample 24 hours after corticotomy, then after three days of corticotomy, pain perception ranged from mild to moderate in 70% of patients and was severe in 20% of patients [6]. In contrast, pain perception was mild in the TCG in the current study during the first four days following corticotomy and continued to decrease over time. The current study also differed from Al-Naoum et al.'s study regarding the discomfort perception variable. In their study, 53.33% of the sample felt moderate to severe discomfort 24 hours after corticotomy, and 50% felt this discomfort after three days [6]. In contrast, discomfort perception in the TCG ranged from mild to moderate during the first four days following corticotomy. The difference may be due to the immediate application of upper canine retraction springs after corticotomy in Al-Naoum et al.'s study, while retraction springs were applied after four days in the current study; this contributes to higher levels of pain and discomfort perception.

The current study also differed from Alsino et al.'s study in that the pain levels were severe 24 hours after the surgical procedure (mean VAS value 80±19.66) [22]; the difference between the current study and Alsino et al.'s study may be attributed to the immediate application of the periodontal accelerated osteogenic orthodontics in the mandibular anterior segments would expectedly cause more pain than in the maxillary spongy bone. The current study agreed with Alfawal et al. that pain levels were mild 24 hours after flapless corticotomy and approached zero after one week [12], differing from Gibreal et al. and Al-Ibrahim et al. and Sirri et al. in that pain levels were mild to moderate 24 hours after flapless corticotomy. The pain levels were mild one week after flapless corticotomy [15,26,27]; this difference between the current study and Gibreal et al., Al-Ibrahim et al., and Sirri et al.'s study may be attributed to the immediate application of flapless corticotomy at the beginning of orthodontic treatment to accelerate tooth movement and alignment, which resulted in pain perception being accompanied by pain resulting from the recent application of fixed orthodontic appliances.

The current study agreed with Alfawal et al. that difficulty chewing and limitation of jaw movement were mild, while difficulty swallowing was almost non-existent 24 hours after flapless corticotomy and continued to decrease over time [12]. The current study differed from Alfawal et al.'s study in the duration of perception of the measured variables, as the perception of difficulty swallowing disappeared after one week, and the rest of the measured variables disappeared after two weeks. In contrast, in the current study, a few patients still had the perception of all measured variables after two weeks. This may be attributed to the fact that the flapless corticotomy was only from the vestibular side in Alfawal et al.'s study, making the surgical intervention less invasive, and Alfawal et al.'s trial was split-mouth designed with the purpose of upper canines’ retraction [12], so the patient's recorded sensation was only for half of the face and may not reflect the full reality if the procedure was applied bilaterally.

When comparing patient satisfaction levels with Alfawal et al.'s study [12], it was found that satisfaction levels in the current study were higher, reaching 97.5%, while in Alfawal et al.'s study, the mean satisfaction levels were 82.94% [12], which may be attributed to the fact that they compared both sides of the mouth, while there was no control group in the current study.

When comparing corticotomy and tooth extraction techniques in terms of discomfort, the current study differed from Al-Naoum et al.'s study [6], which reported that most patients experienced greater discomfort with tooth extraction than with traditional corticotomy. This is contrary to the results of the current study, where 78% of patients reported that traditional corticotomy was more uncomfortable than tooth extraction [6]. This may be attributed to the fact that in their study, corticotomy was performed on one side only, while the other was a control group, whereas tooth extraction was performed in one day and one session. Therefore, corticotomy was more acceptable to their patients, while in the current study, traditional corticotomy was performed on both sides of the maxilla and mandible, resulting in greater pain and discomfort than a tooth extraction.

Regarding flapless corticotomy and tooth extraction, patients in both the current study and Alfawal et al.'s study found tooth extraction to be more uncomfortable [12], with a percentage of 50% in the current study compared to 82.94%. This difference in patient percentage may be attributed to the fact that flapless corticotomy was performed unilaterally in Alfawal et al.'s study [12], while first premolar extractions were performed in a single session, making it more uncomfortable for a higher percentage of patients compared to the current study, where flapless corticotomy was performed bilaterally on both the palatal and vestibular sides. The current study agreed with Alfawal et al.'s study regarding the very high percentage of patients [12], which reached 100%, who reported that they would advise a friend to apply flapless corticotomy in the context of their orthodontic treatment.

Limitations of the current study

There are several limitations in the current study, as there was no blinding of patients during the completion of the questionnaires that expressed their personal opinions and preferences. Thus, the “Hawthorne” effect cannot be eliminated. In addition, there was no control group (without any surgical intervention) to distinguish and differentiate between pain and discomfort resulting from orthodontic treatment and pain and discomfort resulting from corticotomy, whether traditional flapless corticotomy. Finally, the current study relied on comparing pain and discomfort levels between two surgical techniques for accelerating orthodontic movement in Class II Division 1 patients while there are many methods for accelerating tooth movement (such as physical and mechanical methods) with different types of tooth movement (buccal tipping, intrusion, extrusion, and Class III correction) that still need further studies.

## Conclusions

Negative patient-reported outcomes were significantly lower with the flapless corticotomy than with the traditional corticotomy. Patients who underwent traditional corticotomy complained of moderate to severe swelling 24 hours after the surgical intervention. They also reported moderate discomfort, limitations in jaw movement, difficulty chewing, and mild to moderate pain and swallowing difficulty during the first four days following the corticotomy. However, these levels start to decrease in subsequent evaluation periods. At the same time, flapless corticotomy was less problematic and was associated with mild to moderate levels of discomfort, pain, swelling, limitation of jaw movement, and difficulty chewing, with nearly no swallowing difficulty 24 hours after the surgical intervention. Then these levels started to decrease in subsequent evaluation periods and approached zero after one week.

Traditional corticotomy was considered more uncomfortable than tooth extraction as reported in patients in this group, whereas tooth extraction was considered more uncomfortable than flapless corticotomy in the other group. Patient satisfaction, acceptance, and the possibility of recommending the surgical intervention to a friend were significantly greater in the FCG compared to the TCG one.
